# Early short-course treatment fails to prevent disease or relapse upon discontinuance of treatment in infections in addition to COVID-19

**DOI:** 10.1128/jvi.00381-25

**Published:** 2025-05-12

**Authors:** Arthur M. Friedlander

**Affiliations:** 1 US Army Medical Research Institute of Infectious Diseases, Frederick, Maryland, USA; St. Jude Children's Research Hospital, Memphis, Tennessee, USA

**Keywords:** anthrax, pathogenesis, Q fever, COVID-19, rebound

## LETTER

The comprehensive study by Phan et al. (1) modeling viral rebound in COVID-19 patients after discontinuance of early short-course treatment with nirmatrelvir-ritonavir suggests that the mechanisms involve an early and short 5-day treatment course that suppresses an adaptive immune response, coupled with viral persistence and target cell preservation. Similarly, early treatment coupled with persistence of the infecting microorganism after treatment has stopped is suggested as causing disease occurring after stopping antibiotic prophylaxis for bacterial infections such as Q fever and anthrax.

The difference in disease incidence between prophylaxis and early and late antibiotic treatment was observed in human challenge studies with Q fever, which has an incubation period of ~14 days (2). When antibiotic treatment was begun within 24 h of fever and continued for 5–6 days, all 29 volunteers recovered with no relapse. However, when the same regimen was begun within 24 h after exposure, four of five volunteers developed disease upon discontinuance of antibiotics. In a comparable group given the same regimen late in the incubation period, disease was prevented.

In a non-human primate model of inhalational anthrax (3), antibiotic prophylaxis beginning 1–2 hours after infection for 11–15 days prevented disease while on treatment, but 80% (8/10) died when treatment was stopped. This contrasts with animals treated for 10 days beginning after they were bacteremic. Three animals died during treatment, but when treatment was stopped, 100% (7/7) of the animals survived, compared to only 20% (2/10) in the prophylaxis group. The difference in survival after treatment between high mortality in the prophylaxis group and no mortality in the late treatment group was reflected in differences in the acquired immune response between groups. By day 14 after infection, none of the 10 animals in the prophylaxis group developed an immune response compared to 100% (7/7) of surviving animals in the late treatment group. Thus, prophylaxis was associated with high mortality after discontinuance of antibiotics and lack of an immune response, in contrast to the lack of mortality and development of an immune response in the treatment group. This appears similar to the association of early short-duration treatment of COVID-19 and delay of an acquired immune response with viral rebound. In the anthrax model, survival of infecting spores in the host for months after infection resulted in recommendations for a prolonged 60-day course of antibiotics for post-exposure prophylaxis to prevent disease from occurring after discontinuing antibiotics (4). Indeed, combining post-exposure vaccination with antibiotics is recommended to reduce the prolonged antibiotic regimen by inducing an immune response to protect against anthrax after discontinuing antibiotics (4, 5). This is analogous to the use of vaccination and human rabies immune globulin post-exposure to protect against rabies, which has an extended incubation period (6). Thus, in infections with prolonged persistence of the microorganism or incubation periods, early treatment or prophylaxis may suppress the immune response, resulting in disease after treatment stops. Later and/or more prolonged treatment, and in some cases, post-exposure vaccination may be necessary to prevent disease from occurring after treatment is discontinued in other infections as well as in COVID-19.

## EDITOR'S NOTE

**Ed. Note:** The authors of the original article declined to respond.
